# Genetic Interactions Between *P* Elements Involved in piRNA-Mediated Repression of Hybrid Dysgenesis in *Drosophila melanogaster*

**DOI:** 10.1534/g3.114.011221

**Published:** 2014-06-05

**Authors:** Michael J. Simmons, Marshall W. Meeks, Erik Jessen, Jordan R. Becker, Jared T. Buschette, Michael W. Thorp

**Affiliations:** Department of Genetics, Cell Biology, and Development, University of Minnesota, St. Paul, Minnesota 55108-1095

**Keywords:** cytotype regulation, ping-pong cycle, telomere, transposon

## Abstract

Previous studies have shown that telomeric *P* elements inserted at the left end of the *X* chromosome are anchors of the P cytotype, the maternally inherited state that regulates *P*-element activity in the germ line of *Drosophila melanogaster*. This regulation is mediated by small RNAs that associate with the Piwi family of proteins (piRNAs). We extend the analysis of cytotype regulation by studying new combinations of telomeric and nontelomeric *P* elements (*TP*s and non-*TP*s). *TP*s interact with each other to enhance cytotype regulation. This synergism involves a strictly maternal effect, called presetting, which is apparently mediated by piRNAs transmitted through the egg. Presetting by a maternal *TP* can elicit regulation by an inactive paternally inherited *TP*, possibly by stimulating its production of primary piRNAs. When one *TP* has come from a stock heterozygous for a mutation in the *aubergine*, *piwi*, or *Suppressor of variegation 205* genes, the synergism between two *TP*s is impaired. *TP*s also interact with non-*TP*s to enhance cytotype regulation, even though the non-*TP*s lack regulatory ability on their own. Non-*TP*s are not susceptible to presetting by a *TP*, nor is a *TP* susceptible to presetting by a non-*TP*. The synergism between *TP*s and non-*TP*s is stronger when the *TP* was inherited maternally. This synergism may be due to the accumulation of secondary piRNAs created by ping-pong cycling between primary piRNAs from the *TP*s and mRNAs from the non-*TP*s. Maternal transmission of *P*-element piRNAs plays an important role in the maintenance of strong cytotype regulation over generations.

Small RNAs that interact with the Piwi class of proteins—the piRNAs—have been implicated in the regulation of many different families of transposons in the genome of *Drosophila melanogaster* (Brennecke *et al.* 2007, 2008). Among these, the *P*-element family affords an opportunity to dissect this regulatory mechanism genetically and to evaluate it phenotypically. Flies carrying particular *P* elements can be crossed to analyze the components of regulation and to study how these components contribute to the repression of harmful transposon activity. In this article, we investigate interactions between the transposons that anchor *P*-element regulation—located in the telomeric heterochromatin of the *X* chromosome—and interactions between these telomeric transposons and dispersed, nontelomeric transposons that can strengthen regulation.

*P* elements are mobilized by a transposase encoded by structurally complete members of the *P* family (Karess and Rubin 1984). Their activity is normally restricted to the germ line, where it causes hybrid dysgenesis, a syndrome of abnormalities that includes temperature-sensitive sterility and high frequencies of mutation and chromosome breakage (Kidwell *et al.* 1977). These traits occur in the offspring from crosses between P (paternally contributing) males and M (maternally contributing) females, but they are rare or absent in the offspring from crosses between P females and M males, or from P × P or M × M crosses. Flies from P strains have *P* elements in their genomes, but flies from M strains typically do not; those that do are denoted as M′ (Bingham *et al.* 1982). The low frequency of dysgenic traits in the offspring of crosses involving P females indicates that *P* elements are regulated by a maternally transmitted property of P strains. This property, called the P cytotype (Engels 1979), is mediated by *P*-element piRNAs (Brennecke *et al.* 2008).

One locus that produces piRNAs is situated within the telomere-associated sequences (TAS) at the end of the left arm of the *X* chromosome—that is, at the telomere of *X*L (Brennecke *et al.* 2007). The TAS is an array of repeats with variable structure and length. Another array of repeats, distal to the TAS and forming the actual end of *X*L, consists of sequences derived from non-LTR retrotransposons (Mason and Biessmann 1995). Both of these arrays are associated with proteins, including heterochromatin protein 1 (HP1), which is the product of the *Suppressor of variegation 205* [*Su*(*var*)*205*] gene (James *et al.* 1989; Capkova Frydrykova *et al.* 2008). Piwi, the protein encoded by the *piwi* gene, may also be present (Brower-Toland *et al.* 2007; Yin and Lin 2007). The TAS locus produces both sense and antisense piRNAs that match sequences within its repeats; these types of piRNAs have therefore been called repeat-associated small interfering (rasi) RNAs (Vagin *et al.* 2006). If a *P* element has inserted into the TAS, then piRNAs consisting of sense and antisense *P* sequences are also produced (Brennecke *et al.* 2008). The TAS locus, with its inserted *P* element, therefore serves as an anchor of the P cytotype.

Cytotype regulation is established and maintained by *TP*s in the female germ line (Ronsseray *et al.* 1991; Marin *et al.* 2000; Stuart *et al.* 2002; Niemi *et al.* 2004; Simmons *et al.* 2004). Once established, a female can transmit the capacity for regulation to her daughters through the cytoplasm of her eggs—that is, as a strictly maternal effect of the anchoring *TP* (Ronsseray *et al.* 1993; Simmons *et al.* 2007a). This maternal effect implies that regulation is mediated by extrachromosomal factors, presumably piRNAs that were generated by the mother’s *TP*. However, this strictly maternal effect appears to be insufficient to repress *P* transposition in males (Stuart *et al.* 2002; Thorp *et al.* 2009). Cytotype regulation does occur in males, but only if they carry a maternally inherited *TP*. A *TP* that was inherited patroclinously, *i.e.*, from father to son, as in crosses involving females with attached-X chromosomes, does not regulate *P* activity (Niemi *et al.* 2004; Simmons *et al.* 2004). When a male’s *TP* is transmitted to his daughters, as in crosses with females with unattached X chromosomes, its regulatory ability depends on the genotype of the male’s mate (Niemi *et al.* 2004). If the mate comes from an M strain that does not carry a *TP*, then the paternally inherited *TP* has little or no regulatory ability—that is, it is inactive. If the mate is heterozygous for a *TP*, then the paternally inherited *TP* can be activated by an extrachromosomal effect of the mate’s *TP*. This strictly maternal effect has been termed the “pre-P cytotype” (Ronsseray *et al.* 1993) or “presetting” (Niemi *et al.* 2004). Recent analyses suggest that this phenomenon is mediated by maternally inherited piRNAs, and that it is akin to paramutation in plants (De Vanssay *et al.* 2012). If the male’s mate also transmits a *TP* to her daughters, then this *TP* may enhance the reactivation of the paternally inherited *TP* (Niemi *et al.* 2004).

Cytotype regulation anchored in *TP*s can be strengthened by *P* elements at nontelomeric loci even though these non-*TP*s have no regulatory ability on their own (Belinco *et al.* 2009; Simmons *et al.* 2007a, 2012). This synergism is thought to result from a process involving RNAs from the two types of *P* elements. In brief, antisense piRNAs from the *TP*s target and cleave sense RNAs from the non-*TP*s to create a population of sense piRNAs, which in turn target and cleave antisense transcripts from the *TP*s to create more antisense piRNAs. With repetition, this process, called the ping-pong cycle (Aravin *et al.* 2007 Gunawardane *et al.* 2007; Brennecke *et al.* 2007, 2008; Li *et al.* 2009), amplifies the pool of *P*-element piRNAs so that cytotype regulation is strengthened. The enhanced regulatory ability is transmitted through eggs independently of the *TP*s and the non-*TP*s—that is, it is inherited as a strictly maternal effect (Simmons *et al.* 2012).

In this article, we extend the study of genetic interactions between different *TP*s, interactions between *TP*s and non-*TP*s, and how presetting affects these two types of interactions. Several questions are considered. Do two *TP*s interact synergistically to bring about strong cytotype regulation? Can a *TP* interact with or preset a *TP* that has a different DNA sequence? Is synergism between two *TP*s sensitive to mutational depletion of the proteins HP1, Piwi, or Aub [a cytoplasmic member of the Piwi family encoded by the *aubergine* (*aub*) gene]? How does synergism between *TP*s and non-*TP*s compare to synergism between *TP*s? Can a *TP* preset a non-*TP*, and can a non-*TP* preset a *TP*? To answer these questions, we focus on one aspect of dysgenesis, the temperature-sensitive sterility seen in the offspring of crosses between M females and P males. This trait, called gonadal dysgenesis (GD), is due to massive killing of the germ cells by rampant *P*-element activity (Nikki and Chigusa 1986; Khurana *et al.* 2011). Females that carry *TP*s are able to repress GD in at least some of their daughters. Accordingly, we use the frequency of GD to measure the strength of cytotype regulation; a low frequency implies strong regulation. Our analyses demonstrate the importance of genetic interactions between *TP*s and between *TP*s and non-*TP*s in the regulation of the *P*-transposon family.

## Materials and Methods

### Drosophila stocks and husbandry

The genetic materials are described in Lindsley and Zimm (1992), the Flybase website, or references cited in the text. The telomeric *P* elements *TP5* and *TP6* are inserted in one of the repeats within the TAS of chromosome *X*L (Stuart *et al.* 2002), and the telomeric *P* element *NA-P*(1A), here abbreviated *NA*, is inserted at the junction between the retrotransposon array and the TAS of this chromosome (Marin *et al.* 2000). *TP5* and *TP6* have large internal deletions of the 2907-bp-long canonical *P*-element sequence [nucleotides 438–1523 and nucleotides 833–1816, respectively; see Figure 1 of Jensen *et al.* (2008)], and *NA* is deleted for the first 871 bp of this sequence. Consequently, none of these elements encodes the P transposase. These elements are all tightly linked to a mutant allele of the *w* (*white* eyes) locus; for *TP5* and *TP6*, this allele is a null mutation causing bleach white eyes, and for *NA* it is either the null mutation or the *w^sp^* (*white-spotted*) mutation that causes the eyes to be brown. These mutant alleles make it possible to track the *TP*s easily in crosses. Muller-5 Birmingham is a strain with 57 *P* elements in its genome; none is telomeric or capable of producing the P transposase. Collectively, these elements have no intrinsic ability to repress gonadal dysgenesis; thus, Muller-5 Birmingham is a strain (Simmons *et al.* 2007a). *H*(*hsp*/*TP5*)*D* is a *hobo* transgene containing a cloned *TP5* element that is terminally truncated to prevent it from being mobilized by the P transposase (Jensen *et al.* 2008). The promoter of this element is augmented with a heat-shock-inducible promoter from the *hsp70* gene of *Drosophila*; however, this transgene is expressed in the absence of heat shocks, and no heat shocks were used in the experiments reported here. The *H*(*hsp*/*TP5*)*D* transgene is marked with a wild-type allele of the *white* gene, which confers pigment in the eyes. Stock cultures were reared on a cornmeal-molasses-dried yeast medium in vials or half-pint milk bottles at 18° or 21°. Experimental cultures were incubated at 25° or at temperatures specified in the text.

### Assay for gonadal dysgenesis

Gonadal dysgenesis (GD) occurs when *P* elements inherited from males of a strong P strain such as Harwich *y w* (Simmons *et al.* 2012) are activated in the germ line of a developing zygote, killing the germ-line cells and causing the adult to be sterile. GD can be repressed by maternally transmitted factors. To test females for their ability to produce these factors, we mated them *en masse* to Harwich *y w* males at 21° and then transferred each mated female to a separate culture 3 d later. These single-female cultures were incubated at 29°, a temperature that maximizes GD; on day 11, the progeny from each culture were transferred to a holding vial, which was kept at 21° for 2 d to allow the progeny to mature. Samples of the matured females were examined for eggs by squashing them between two glass slides; green food coloring was placed between the slides to make the eggs easier to see. Females that did not produce eggs were classified as having GD. When cultures segregated females with different genotypes, the genotypes were scored separately, unless noted otherwise; no more than 20 females of each genotype were examined from each culture. The crosses that produced the females for test crosses to Harwich *y w* males were incubated at 25°. Details of these crosses are presented in *Results*.

### Statistical analyses

We calculated the percentage of females with GD in each vial and then computed the unweighted average percentage of GD for all the vials in the test group. The SE of this average was obtained from the associated empirical variance. Differences between averages were evaluated by performing *t* or *z* tests.

## Results

### Synergism between homozygous telomeric *P* elements

Previous studies have not directly addressed if cytotype regulation is enhanced by synergistic interactions between two *X*-linked *TP*s. To investigate this issue, we performed test crosses between females that were homozygous or heterozygous for particular *TP*s and males from the Harwich *y w* P strain and examined their daughters for gonadal dysgenesis (Table 1). We also included test crosses with females from two M strains that did not carry a *TP* (or any other *P* element).

**Table 1:** Gonadal dysgenesis in the daughters of females homozygous or heterozygous for a *TP*

				Females Heterozygous for *TP*					
	Females Homozygous for *TP*			*TP* Initially Derived from Females (Cross A)*^a^*			*TP* Initially Derived from Males (Cross B)*^b^*		
*TP*	No. Vials	No. Flies	%GD ± SE*^c^*	No. Vials	No. Flies	%GD ± SE*^c^*	No. Vials	No. Flies	%GD ± SE*^c^*
None (Sam)	11	81	99.3 ± 0.7						
None (*w*)	10	83	100	30	576	99.3 ± 0.4	30	550	99.8 ± 0.2
*TP5**^d^*	28	373	13.7 ± 3.8	39	971	94.6 ± 0.9	33	783	98.2 ± 0.7
*TP6**^d^*	27	281	32.5 ± 4.6	37	855	78.2 ± 2.2	34	769	96.8 ± 0.7
*NA**^e^*	17	145	0	39	993	64.0 ± 2.7	32	678	99.0 ± 0.4

Gonadal dysgenesis was assessed in the daughters of test crosses between females homozygous or heterozygous for a *TP* and Harwich *y w* males. In crosses that segregated different genotypes, the daughters with the *TP* and those without it were scored separately, but because there were no differences between them, the results have been pooled.

a In cross A, *TP/TP* females were mated to wild-type males from the M strain Samarkand (Sam), which is devoid of *P* elements, to produce *TP*/+ heterozygotes.

b In cross B, *TP* males were mated to wild-type females from the M strain Samarkand to produce +/*TP* heterozygotes.

c Unweighted average percentage GD ± SE.

d *TP5* and *TP6* are tightly linked to a null allele of the *w* locus.

e *NA* is tightly linked to the *w^sp^* allele of the *w* locus.

Almost all (>99%) of the daughters from the M females were dysgenic, demonstrating that Harwich *y w* is a powerful inducer of GD. In contrast, most of the daughters of the *TP* homozygotes were not dysgenic, showing that they could repress the activity of the Harwich *P* elements. The *NA* and *TP5* homozygotes were the strongest repressors, with 0% and 13.7% dysgenic daughters, respectively. The *TP6* homozygotes, with 32.5% dysgenic daughters, repressed GD less strongly.

The *TP* heterozygotes that we studied came from two sets of crosses. In one set, homozygous *TP* females were mated to males from an M strain (cross A); in the other set, females from the M strain were mated to *TP* males (cross B)—that is, the A and B types of heterozygotes came from reciprocal crosses between the *TP* and M strains. These two types of heterozygotes were genetically (*i.e.*, chromosomally) identical. However, they differed in the extrachromosomal factors that are transmitted through the egg cytoplasm. The cross B heterozygotes did not repress GD in their daughters, whereas the cross A heterozygotes did—a difference indicating that the paternally inherited *TP*s in the cross B heterozygotes are inactive. Among the cross A heterozygotes, those carrying either *TP6* or *NA* were moderate repressors (78.2% GD and 64.0% GD, respectively), whereas those carrying *TP5* were very weak repressors (94.6% GD). Repression by the cross A heterozygotes was seen equally in the daughters that carried a *TP* and in those that did not. Thus, the repression was mediated by a strictly maternal (*i.e.*, extrachromosomal) effect. None of the *TP* heterozygotes from cross A repressed GD as well as the *TP* homozygotes did. Doubling the dose of a *TP* therefore strengthens cytotype regulation significantly. The effect of the doubled dose is much greater than the doubled effect of a single maternally inherited *TP*. Strong regulation of *P*-element activity therefore involves synergism between the two elements in a *TP* homozygote.

### Synergism between combinations of different telomeric *P* elements

**Figure 1:**
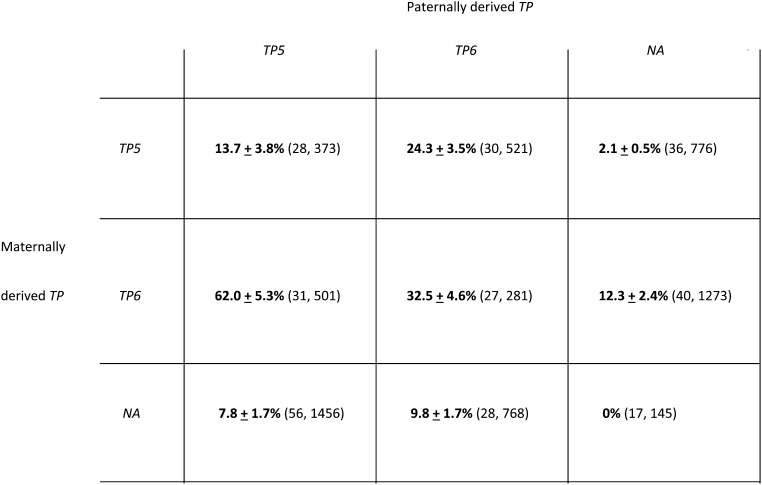
Gonadal dysgenesis in the daughters of females carrying combinations of *TP*s. These combinations were produced by performing reciprocal crosses between different *TP* strains at 25°. Data from *TP* homozygotes (from Table 1), which were tested at the same time, are included for comparison. The unweighted average percentages of GD ± SE are in boldface. The numbers of test vials and daughters examined are in parentheses. The *TP5* and *TP6* elements were tightly linked to a null allele of the *w* locus, and the *NA* element was linked to the *w^sp^* allele of this locus. In test crosses where the *w* and *w^sp^* alleles segregated (*e.g.*, *NA w^sp^/TP5 w* females × Harwich *y w* males), the two classes of daughters were scored separately; however, in all such test crosses the results have been pooled because there were no differences between them.

Previous studies have not determined if two different *TP*s can interact to enhance cytotype regulation. To address this issue, we created combinations of *TP*s by performing reciprocal crosses between each of the *TP* strains and then tested these combinations for their ability to repress GD in the next generation (Figure 1). Females with combinations of *NA* and *TP5* or *NA* and *TP6* had a strong ability to repress GD in their offspring, no matter how the *TP*s were combined (≤12.3% GD). Thus, the *NA* element was able to interact genetically with either *TP5* or *TP6* to bring about strong regulation of *P*-element activity. By contrast, the two types of *TP5*/*TP6* combinations had different abilities to repress GD—a strong ability (24.3% GD) when the *TP5* element was maternally derived *vs.* a moderate ability (62.0% GD) when it was paternally derived. This difference indicates that a maternal effect can influence the genetic interaction between two telomeric *P* elements.

### Presetting effects of telomeric *P* elements on cytotype regulation by paternally inherited telomeric *P* elements

**Figure 2:**
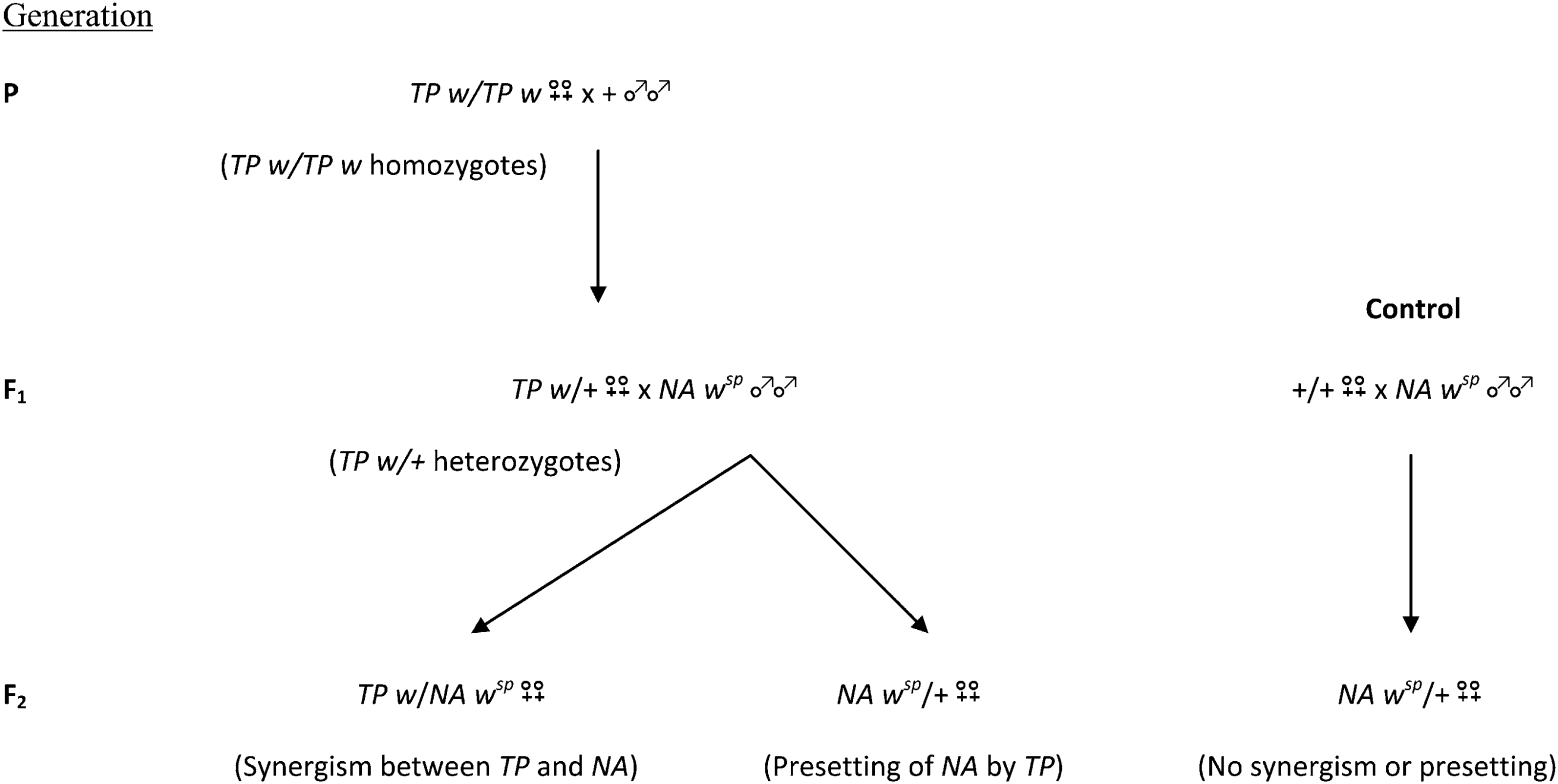
Scheme to test for presetting of a paternally inherited *TP* by another *TP*. The presetting elements *TP5*, *TP6*, and *NA* originated in the females of the P generation; each of these elements was tightly linked to a null allele of the *w* locus. In the crosses involving *TP5* and *TP6*, these females were homozygotes, whereas in the cross involving *NA*, they were *NA w/FM7* heterozygotes. The target of presetting by these *TP*s was an *NA* element linked to the *w^sp^* allele. The wild-type flies that were used in the initial crosses came from the M strain Samarkand. The different eye color markers made it possible to track the inheritance of the various telomeric *P* elements throughout the experiment. All the crosses in this scheme were incubated at 25°; however, test crosses between the various types of females and Harwich *y w* males were performed as described in the *Materials and Methods*.

Previous studies have shown that the strictly maternal (presetting) effects of *TP5*, *TP6*, and *NA* can enhance regulation by a paternally inherited *TP* (Marin *et al.* 2000; Niemi *et al.* 2004). To extend these studies, we performed an experiment to test if maternally transmitted factors from heterozygous *TP5 w*/+, *TP6 w*/+, or *NA w*/+ females could enhance the regulatory ability of a paternally inherited *NA* element linked to *w^sp^* (Figure 2). In each generation, samples of females were test-crossed to Harwich *y w* males and their daughters were scored for GD. The results (Table 2) show that all the F_1_ *TP w*/+ heterozygotes enhanced the regulatory capacity of the paternally inherited *NA* element through a strictly maternal (*i.e.*, presetting) effect. In the F_2_, the control *NA w^sp^*/+ females, whose +/+ mothers did not carry a potentially presetting *TP*, had negligible ability to repress GD in their daughters (97.6% GD). By contrast, the *NA w^sp^*/+ females whose F_1_ mothers carried a potentially presetting *TP* were able to repress GD in their daughters. This repression was most pronounced when the presetting element was *TP5* (52.5% GD), but it was also statistically significant when the presetting element was either *TP6* or *NA* (88.0% GD and 92.1% GD, respectively). The other data in Table 2 document the regulatory properties of the various *TP*s in different situations. As expected, the *TP* homozygotes were moderate to strong repressors of GD in their daughters, the *TP w*/+ heterozygotes were weak to moderate repressors, and the *TP*/*NA* combinations were very strong repressors. From this experiment, we see that presetting by maternally transmitted factors can play a role in the emergence of strong cytotype regulation in females that carry two *TP*s.

**Table 2:** Gonadal dysgenesis in the daughters of test crosses to detect the presetting effects of *TP*s on the telomeric element *NA*

	*TP w/TP w* Homozygotes			F_1_ *TP w*/+ Heterozygotes*^a^*			F_2_ Synergism*^b^*			F_2_ Presetting Effect*^c^*		
*TP*	No. Vials	No. Flies	%GD ± SE*^d^*	No. Vials	No. Flies	%GD ± SE*^d^*	No. Vials	No. Flies	%GD ± SE*^d^*	No. Vials	No. Flies	%GD ± SE*^d^*
*NA w^sp^*	25	360	4.0 ± 1.0									
None (+)*^e^*	25	335	99.2 ± 0.8							27	567	97.6 ± 0.8*^f^*
*TP5 w*	20	397	8.3 ± 1.6	32	798	67.4 ± 3.3	25	587	0.7 ± 0.4	28	841	52.5 ± 4.5
*TP6 w*	24	367	57.6 ± 5.5	32	761	70.6 ± 3.2	28	712	3.6 ± 0.9	28	948	88.0 ± 2.0
*NA w**^g^*				28	765	98.2 ± 0.8	30	782	5.3 ± 1.7	30	844	92.1 ± 2.1

Gonadal dysgenesis was assessed in the daughters of test crosses between the various types of females obtained through the scheme in Figure 2 and Harwich *y w* males. In segregating crosses, different genotypes were scored separately, but because there were no differences between them, the results have been pooled.

a These F_1_ heterozygotes were obtained by crossing *TP w/TP w* homozygotes to + males from the M strain Samarkand, except in the case of *NA w*, where the cross was *NA w/FM7* females × + males.

b Synergism between a *TP* and the *NA* element was assessed by testing *TP w/NA w^sp^* F_2_ females from crosses between *TP w*/+ F_1_ heterozygotes and *NA w^sp^* males (see Figure 2).

c The presetting effect of a *TP* on the *NA* element was assessed by testing +/*NA w^sp^* F_2_ females from crosses between *TP w*/+ F_1_ heterozygotes and *NA w^sp^* males (see Figure 2).

d Unweighted average percentage GD ± SE.

e The wild-type flies came from the M strain Samarkand, which is devoid of *P* elements.

f These data were obtained from tests with the +/*NA w^sp^* daughters of crosses between Samarkand (+) females and *NA w^sp^* males—that is, from the control cross in Figure 2.

g Females homozygous for the *NA w* chromosome produce many eggs that do not hatch, a form of sterility that is unrelated to hybrid dysgenesis. Consequently, this chromosome was maintained with the *FM7* balancer in heterozygous condition, which may explain why the presetting effect of this *NA* element (see rightmost column) on a paternally inherited *NA* element is so weak

### Mutational disruption of synergism between two telomeric *P* elements

Previous studies have implicated the proteins encoded by the *aub*, *piwi*, and *Su*(*var*)*205* genes as important factors in cytotype regulation (Ronsseray *et al.* 1996; Reiss *et al.* 2004; Haley *et al.* 2005; Josse *et al.* 2007; Simmons *et al.* 2007b, 2010). However, these studies have not addressed if mutational depletion of these proteins disrupts the synergism between two *TP*s. To investigate this issue, we assessed the regulatory abilities of *TP5/NA* females whose *TP5* element came from a stock that was heterozygous for an *aub*, *piwi*, or *Su*(*var*)*205* mutation; the tested females were also heterozygous for this mutation. As controls, we used stocks that were heterozygous for *Gla*, a mutation that has not been implicated in any aspect of cytotype regulation.

The end-points in this experiment were the GD frequencies among the daughters of the tested females. These frequencies could reflect the immediate effect of the mutation in the female’s genotype, or a cumulative (multi-generational) effect of the mutation in the stock from which the *TP5* element and the mutation were derived. The different female genotypes were created in three sets of crosses. In cross 1, the *TP5* element (linked to a null allele of *w*) and the mutation were maternally inherited, whereas in crosses 2 and 3, they were paternally inherited. The *NA* element (linked to the *w^sp^* allele) that could interact genetically with *TP5* was inherited paternally in cross 1 and maternally in crosses 2 and 3; however, in cross 3, the *NA* element was transmitted from heterozygous rather than homozygous mothers—a condition that might diminish its regulatory ability. Thus, cross 3 provided an opportunity to assess the effects of the various mutations in *TP5/NA* females that might be more sensitive to these effects. The results of all the test crosses are summarized in Table 3.

**Table 3:** Effects of mutations on repression of gonadal dysgenesis by synergism between the telomeric elements *TP5* and *NA*

	Cross 1 (*TP5 w*; *mut*/*CyO* ♀♀ × *NA w^sp^* ♂♂)			Cross 2 (*NA w^sp^* ♀♀ × *TP5 w*; *mut/CyO* ♂♂)			Cross 3 (*NA w^sp^*/*y w* ♀♀ × *TP5 w*; *mut/CyO* ♂♂)		
Mutation*^a^*	No. Vials	No. Flies	%GD ± SE*^b^*	No. Vials	No. Flies	%GD ± SE*^b^*	No. Vials	No. Flies	%GD ± SE*^b^*
*Gla* (control)	25	592	0.5 ± 0.3	21	506	4.9 ± 0.7	30	494	33.5 ± 4.9
*Gla* (no *TP5*)*^c^*	27	412	95.9 ± 1.2	27	706	66.5 ± 3.5	14	177	98.1 ± 1.3
*Gla* (no *NA*)*^d^*	25	589	97.1 ± 0.7	29	556	99.0 ± 0.4			
*aub^QC42^*	27	767	42.0 ± 6.3	26	772	4.9 ± 0.8	25	484	35.0 ± 6.5
*aub^∆P-3a^*	25	511	25.5 ± 4.0	28	833	13.3 ± 1.6	25	433	43.0 ± 6.2
*piwi^1^*	27	634	3.3 ± 1.4	27	808	12.0 ± 1.6	25	474	79.7 ± 4.1
*piwi^2^*	27	474	3.5 ± 1.0	23	475	19.3 ± 5.9	25	493	80.7 ± 3.0
*Su*(*var*)*205^4^*	22	533	96.0 ± 2.4	27	900	89.6 ± 2.0	25	472	94.8 ± 2.2

Gonadal dysgenesis was assessed in the daughters of *TP5 w/NA w^sp^*; *mutation*/+ F_1_ females obtained from crosses 1, 2, and 3. With the F_1_ females from crosses 1 and 2, the *TP5 w*-bearing and *NA w^sp^*-bearing daughters were scored separately, but because there were no differences between them, the results have been pooled. With the F_1_ females obtained from cross 3, the *TP5 w*-bearing and *NA w^sp^*-bearing daughters were lumped together for scoring. The *NA w^sp^/y w* females for cross 3 were obtained by crossing *NA w^sp^* females with *y w* males from an M strain devoid of *P* elements.

a The mutant stocks are described by Belinco *et al.* (2009). The *TP5* element in all these stocks was derived from the *TP5 w*; *Gla*/*CyO* control stock.

b Unweighted average percentage GD ± SE.

c The *TP5 w*; *Gla/CyO* flies in crosses 1 and 2 were replaced by *w*; *Gla/CyO* flies; hence, the females tested were *w/NA w^sp^*; *Gla*/+. The *w*-bearing and *NA w^sp^*-bearing daughters were scored separately, but the results have been pooled.

d The *NA w^sp^* flies in crosses 1 and 2 were replaced by wild-type flies from the M strain Samarkand; hence, the females tested were *TP5 w*/+; *Gla*/+. The females that carried *TP5* w and those that did not were scored separately, but the results have been pooled.

The *Gla* control at the top of Table 3 shows that synergism between *TP5* and *NA* in crosses 1 and 2 led to very strong repression of dysgenesis (<5% GD). In cross 3, this repression was not as strong (33.5% GD), indicating that, as hypothesized, the synergism between *NA* and *TP5* is weakened when the *NA* element is inherited from heterozygous mothers. The *Gla* control in which *TP5* was absent shows that by itself, a heterozygous *NA* element inherited from homozygous mothers leads to moderate repression (66.5% GD), but when inherited paternally or from heterozygous mothers, its repression ability is negligible (≥95.9% GD). The *Gla* control in which *NA* was absent shows that by itself, a heterozygous *TP5* element inherited paternally or from homozygous mothers has negligible repression ability (≥97.1% GD).

Among the mutations tested, *Su*(*var*)*205^4^* had the greatest impact on synergism between *TP5* and *NA*. In all three crosses, this mutation profoundly disrupted the ability of the *TP5/NA* females to repress GD in their daughters (≥89.6% GD). This telling effect is consistent with published data showing that *Su*(*var*)*205^4^* significantly impairs regulation by a single *TP* (Ronsseray *et al.* 1998; Marin *et al.* 2000; Haley *et al.* 2005; Belinco *et al.* 2009; Simmons *et al.* 2010). The *aub* and *piwi* mutations had less detrimental effects. Both mutant *aub* alleles moderately weakened cytotype regulation in the *TP5/NA* females from cross 1 (≥25.5% GD compared with the control value 0.5%)—that is, when the *TP5* element and the *aub* mutation were inherited maternally, but they had much smaller effects in the females from cross 2 (≤13.3% GD compared with the control value 4.9%) or cross 3 (<43.0% GD compared with the control value 33.5%), where *TP5* and the *aub* mutation were inherited paternally. These results indicate that the *aub* mutations impair synergism between two *TP*s through a maternal effect. The *piwi* mutations had little or no detrimental effects on the synergism between *TP5* and *NA* in crosses 1 and 2; however, in cross 3, where the *NA* element came from heterozygous mothers, these mutations significantly impaired regulation by the *TP5/NA* combination (80% GD compared with the control value 33.5%). Thus, the *piwi* mutations impair regulation through a zygotic effect in *TP5/NA* females that already have a diminished capacity for regulation because they inherited their *NA* element from heterozygous mothers.

### Synergism between telomeric and nontelomeric *P* elements

The telomeric elements *TP5* and *TP6* interact genetically with nontelomeric *P* elements to bring about very strong cytotype regulation (Simmons *et al.* 2007a, 2012). To see if regulation by the telomeric element *NA* could also be strengthened by genetic interactions with nontelomeric *P* elements, we combined this element with the numerous nontelomeric *P* elements on the autosomes of the M′ strain Muller-5 Birmingham, here denoted simply as *Birm*. The procedure was to perform reciprocal crosses between the *NA* and *Birm* strains: *NA* females × *Birm* males (cross A), and *NA* males × *Birm* females (cross B). The F_1_ daughters of these crosses were then test-crossed to Harwich y w males to produce F_2_ females that were scored for GD according to whether they inherited the *NA* element, which was tightly linked to the *w^sp^* marker. For controls, we produced *NA w^sp^*/+ F_1_ females by reciprocally crossing *NA w^sp^* flies to flies from the M strain Samarkand, and we produced *Birm*/+ F_1_ females by reciprocally crossing flies from the Samarkand and *Birm* strains. The control F_1_ females from these pairs of reciprocal crosses were then crossed to Harwich *y w* males to induce GD in the F_2_. The results from this experiment (Table 4) warrant several conclusions.

**Table 4:** Synergistic repression of gonadal dysgenesis by the telomeric *P* element *NA* and the nontelomeric autosomal *P* elements from Muller-5 Birmingham

		*NA* Present in F_2_		*NA* Absent in F_2_		Pooled Overall	
Reciprocal Crosses to Produce F_1_ Females for Testing	No. Vials	No. Flies	%GD ± SE*^a^*	No. Flies	%GD ± SE*^a^*	No. Flies	%GD ± SE*^a^*
A: + female × *Birm* male	25					405	100 ± 0
B: *Birm* female × + male	22					381	100 ± 0
A: *NA* female × + male	23	213	46.0 ± 4.7	199	52.7 ± 4.4	412	47.6 ± 3.7
B: + female × *NA* male	27	210	92.7 ± 0.5	201	99.5 ± 0.5	411	95.6 ± 1.0
A: *NA* female × *Birm* male	25	231	1.0 ± 0.5	246	3.0 ± 1.2	477	2.1 ± 0.7
B: *Birm* female × *NA* male	27	224	38.7 ± 5.3	185	47.3 ± 5.6	409	41.6 ± 4.7

Gonadal dysgenesis was assessed in the F_2_ daughters of F_1_ females produced by the reciprocal crosses shown (see text for details). The F_2_ females that did or did not carry the *NA* element (closely linked to the *w^sp^* marker) were scored separately.

a Unweighted average percentage GD ± SE.

First, because 100% of the offspring of both types of control *Birm*/+ F_1_ females had GD, the Harwich *y w* strain is a powerful inducer of GD and the *Birm P* elements are unable to repress this GD. Second, the *NA* element is able to repress GD, especially when the *NA w^sp^*/+ F_1_ females inherited *NA* maternally—that is, through cross A. The daughters of these females had much less GD (47.6%) than those derived from cross B (95.6%). Third, repression of GD is enhanced when *NA* acts in combination with the *Birm P* elements. In cross A, when *NA* acted alone, 47.6% of the F_2_ females had GD, whereas when it acted together with the *Birm P* elements, only 2.1% of them had GD. In cross B, when *NA* acted alone, 95.6% of the F_2_ females had GD, whereas when it acted together with the *Birm P* elements, 41.6% of them had GD. The *NA* and *Birm P* elements therefore interact synergistically to regulate *P* activity, even when the *NA*/+; *Birm/+* F_1_ females had inherited the *NA* element paternally. Fourth, *NA*-mediated regulation occurs in F_2_ females even when they do not inherit the *NA* element itself. In the tests with the *NA w^sp^*/+ F_1_ females from cross A, GD was repressed almost as well in the F_2_ daughters that did not carry *NA* (52.7% GD) as in those that did (46.0% GD). In the tests with the *NA w^sp^*/+; *Birm*/+ F_1_ females from cross A, GD was repressed almost completely in both classes of daughters, and in the tests with the *NA w^sp^*/+; *Birm*/+ F_1_ females from cross B, GD was repressed partially in both classes of F_2_ daughters (38.7% GD in those with *NA* and 47.3% GD in those without *NA*). Repression of GD is therefore mediated by a maternal effect established by *NA*, or by an interaction between *NA* and the *Birm P* elements, in the F_1_ females. The strength of this effect depends on whether an F_1_ female inherited *NA* maternally (moderate to strong repression) or paternally (weak to moderate repression), and on whether the *Birm P* elements were present (moderate to strong repression) or absent (weak to moderate repression) in the F_1_ female. Thus, the *NA* element has the same regulatory characteristics as the previously studied telomeric elements *TP5* and *TP6* (Simmons *et al.* 2007a; Belinco *et al.* 2009).

### Absence of presetting effects on and by nontelomeric *P* elements

Presetting by a maternal *TP* can enhance the regulatory ability of a paternally inherited *TP*. However, presetting by a maternal *TP* apparently does not enhance regulation by paternally inherited non-*TP*s (Belinco *et al.* 2009). We re-examined this issue by performing an experiment with the telomeric *P* elements *TP5*, *TP6*, and *NA* and the nontelomeric *P* elements of the M′ strain Muller-5 Birmingham, here denoted *M5*; *Birm*; this same strain has previously been used to study interactions between *TP*s and non-*TP*s (Simmons *et al.* 2007a; Belinco *et al.* 2009). The scheme for the experiment is outlined and the results are summarized in Table 5. Several conclusions can be drawn from these results. First, GD was not repressed in the M controls (groups 1–3), nor when the Birmingham *P* elements acted alone (groups 4 and 5). Second, GD was repressed slightly or moderately by the *TP*s acting alone (groups 6 and 7, 11 and 12, and 16 and 17). *NA* had the greatest regulatory ability in these tests—64.2% GD in the F_1_ and 80.4% GD in the F_2_; *TP6* had the next greatest—80.1% GD in the F_1_ and 96.6% GD in the F_2_; and *TP5* had the least regulatory ability—96.2% GD in the F_1_ and 91.9% GD in the F_2_. GD was not repressed when these *TP*s were removed from the F_2_ genotype (groups 8, 13, and 18). Third, GD was repressed strongly by the *TP*s in combination with the nontelomeric Birmingham *P* elements (groups 9, 14, and 19), but it was not repressed at all by Birmingham *P* elements that had been exposed to the presetting effects of these *TP*s (groups 10, 15, and 20). Thus, collectively, the non-*TP*s in the *M5*; *Birm* strain are not susceptible to presetting by the *TP5*, *TP6*, or *NA* telomeric elements.

**Table 5:** Gonadal dysgenesis in the daughters of test crosses to detect the presetting effects of *TP*s on the *P* elements in Muller-5 Birmingham

Test Group	F_1_ Females*^a^*^,^*^c^* ×	F_1_ Males*^b^* →	F_2_ Females*^c^*	No. Vials	No. Flies	%GD ± SE*^d^*	Issue Tested
1	*w*/+			25	456	100	M strain control
2		*w*	*w/w*	20	341	100	M strain control
3			+/*w*	25	447	100	M strain control
4		*M5*; *Birm*	*w*/*M5*; +/*Birm*	25	500	99.8 ± 0.2	Effect of Birmingham *P* elements alone
5			+/*M5*; +/*Birm*	25	491	100	Effect of Birmingham *P* elements alone
6	*TP5 w*/+			25	500	96.2 ± 1.2	Effect of *TP5* alone in F_1_
7		*w*	*TP5 w*/*w*	25	441	91.9 ± 1.9	Effect of *TP5* alone in F_2_
8			+/*w*	25	469	100	Effect of removing *TP5* in F_2_
9		*M5*; *Birm*	*TP5 w*/*M5*; +/*Birm*	27	459	27.1 ± 3.9	Synergism between *TP5* and Birmingham *P* elements
10			+/*M5*; +/*Birm*	25	490	100	Presetting effect of *TP5* on Birmingham *P* elements
11	*TP6 w*/+			25	291	80.1 ± 2.7	Effect of *TP6* alone in F_1_
12		*w*	*TP6 w/w*	23	279	96.6 ± 1.2	Effect of *TP6* alone in F_2_
13			+/*w*	25	379	100	Effect of removing *TP6* in F_2_
14		*M5*; *Birm*	*TP6 w/M5*; +/*Birm*	25	492	7.1 ± 2.6	Synergism between *TP6* and Birmingham *P* elements
15			+/*M5*; +/*Birm*	25	419	100	Presetting effect of *TP6* on Birmingham *P* elements
16	*NA w^sp^*/+			25	434	64.2 ± 3.4	Effect of *NA* alone in F_1_
17		*w*	*NA w^sp^/w*	25	465	80.4 ± 2.6	Effect of *NA* alone in F_2_
18			+/*w*	25	455	100	Effect of removing *NA* in F_2_
19		*M5*; *Birm*	*NA w^sp^/M5*; +/*Birm*	25	410	18.5 ± 3.1	Synergism between *NA* and Birmingham *P* elements
20			+/*M5*; +/*Birm*	25	414	99.5 ± 0.3	Presetting effect of *NA* on Birmingham *P* elements

Four different types of F_1_ females that were heterozygous for a *TP* (or not, in the case of the controls) and a mutant *w* allele were crossed with two different types of F_1_ males to produce the various types of F_2_ females that were test-crossed to Harwich *y w* males. Samples of each of the four types of F_1_ females were also test-crossed with Harwich *y w* males. The daughters of all the test crosses were scored for GD without being sorted by genotype.

a F_1_ females were obtained by crossing females homozygous for a *TP* (or not, in the case of the controls) to wild-type males from the M strain Samarkand.

b F_1_ males came either from an M strain marked with a null allele of *w* or from the M’ strain Muller-5 Birmingham. The *Muller-5* (*M5*) balancer X chromosome in this latter strain is marked with *w**^a^* and *B*; the autosomal *P* elements in this strain are symbolized as *Birm*.

c In these heterozygous genotypes, the maternally inherited components are written on the left side of the slashes.

d Unweighted average percentage GD ± SE.

Another experiment determined if the regulatory ability of an individual non-*TP* could be influenced by presetting. This non-*TP* was a cloned version of *TP5* contained within a *hobo* transgene inserted at map position 73.6 in the middle of chromosome *2*R. The transgene, denoted *H*(*hsp*/*TP5*)*D*, is marked with a *w*^+^ allele and has no intrinsic ability to repress GD; however, it and other insertions of H(*hsp*/*TP5*) can interact genetically with *TP5*, *TP6*, or *NA* to enhance cytotype regulation significantly (Simmons *et al.* 2012; Jessen *et al.* 2013). We determined if paternally inherited transgenic and telomeric *TP5* elements were susceptible to the presetting effects of the telomeric elements *TP5* and *NA* (Table 6).

**Table 6:** Gonadal dysgenesis in the daughters of test crosses to detect the presetting effects of *TP*s with a transgenic *P* element

Test Group	F_1_ Females*^a^*^,^*^c^* ×	F_1_ Males*^b^* →	F_2_ Females*^c^*	No. Vials	No. Flies	%GD ± SE*^d^*	Issue Tested
0	*y w/TP5 w*			23	252	100	Repression by paternally inherited telomeric *TP5* element
1	*TP5 w/y w*	*y w*	*TP5 w/y w*	25	394	92.8 ± 1.7	Repression by maternally inherited telomeric *TP5* element
2			*y w/y w*	25	459	100	Repression by cytoplasm from *TP5 w/y w* F_1_ females
3		*TP5 w*	*TP5 w/TP5 w**^e^*	21	236	14.6 ± 3.4	Synergism between two telomeric *TP5* elements
4			*y w/TP5 w**^e^*	23	337	88.0 ± 2.5	Presetting of one telomeric *TP5* element by another
5		*y w*; *H*(*hsp*/*TP5*)*D*	*TP5 w*/*y w*; *H*(*hsp*/*TP5*)*D*/+*^f^*	25	247	6.9 ± 1.7	Synergism between telomeric *TP5* and transgenic *TP5* elements
6			*y w/y w*; *H*(*hsp*/*TP5*)*D*/+*^f^*	25	283	100	Presetting of transgenic *TP5* element by telomeric *TP5* element
7	*NA w^sp^/y w*	*y w*	*NA w^sp^/y w*	25	724	78.9 ± 2.5	Repression by maternally inherited telomeric *NA* element
8			*y w/y w*	25	473	100	Repression by cytoplasm from *NA w^sp^/y w* F_1_ females
9		*TP5 w*	*NA w^sp^/TP5 w*	25	681	5.1 ± 1.4	Synergism between telomeric *NA* and telomeric *TP5* elements
10			*y w/TP5 w*	25	777	86.4 ± 1.8	Presetting of telomeric *TP5* element by telomeric *NA* element
11		*y w*; *H*(*hsp*/*TP5*)*D*	*NA w^sp^/y w*; *H*(*hsp*/*TP5*)*D*/+	25	871	13.9 ± 4.0	Synergism between telomeric *NA* and transgenic *TP5* elements
12			*y w/y w*; *H*(*hsp*/*TP5*)*D*/+	25	698	100	Presetting of transgenic *TP5* element by telomeric *NA* element
13	*w*; *H*(*hsp*/*TP5*)*D*/+	*TP5 w*	*w*/*TP5w*; +/+	19	173	98.8 ± 0.6	Presetting of telomeric *TP5* element by transgenic *TP5* element
14			*w*/*TP5 w*; *H*(*hsp*/*TP5*)*D*/+	6	51	100	Synergism between maternally inherited transgenic *TP5* element and paternally inherited telomeric *TP5* element

Gonadal dysgenesis was assessed in the daughters of test crosses between F_1_ females from group 0 and Harwich *y w* males and in the daughters of test crosses between F_2_ females from groups 1–14 and Harwich *y w* males. Except where noted, data from the genotypes that segregated in the test crosses have been pooled.

a For group 0, F_1_ females were obtained by crossing *y w* females from an M strain devoid of *P* elements to *TP5 w* males. For groups 1–6, F_1_ females were obtained by crossing homozygous *TP5 w* females to *y w* males from this M strain. For groups 7–12, F_1_ females were obtained by crossing homozygous *NA w^sp^* females to *y w* males from this M strain. The *y*^+^ allele present in the *TP5 w* and *NA w^sp^* chromosomes is not shown. For groups 13 and 14, F_1_ females were obtained by crossing homozygous *w* females from an M strain devoid of *P* elements to *w*; *H*(*hsp*/*TP5*)*D* males.

b F_1_ males came from the *y w* M strain, the *TP5 w* strain, or the *y w* strain carrying the *H*(*hsp*/*TP5*)*D* transgene.

c In these heterozygous genotypes, the maternally inherited components are written on the left side of the slashes.

d Unweighted average percentage GD ± SE.

e These two types of females were distinguished by whether they produced yellow-bodied offspring when test-crossed to Harwich *y w* males.

f All the F_3_ females that were scored carried the *H*(*hsp*/*TP5*)*D* transgene.

A paternally inherited telomeric *TP5* element had no ability to repress GD (group 0). However, this element did acquire weak, but statistically significant, repression ability when exposed to the presetting effects of either the *TP5* (88.0% GD, group 4) or the *NA* (86.4% GD, group 10) telomeric elements. Both of these telomeric elements had some repression ability when they were maternally inherited; *TP5* (group 1) repressed GD slightly (92.8%) and *NA* (group 7) repressed GD moderately (78.9%). However, when these elements were absent from the F_2_ genotype, GD was not repressed at all (groups 2 and 8). As expected, F_2_ females that carried two telomeric *P* elements repressed GD strongly—14.6% GD when the females carried two *TP5* elements (group 3) and 5.1% GD when they carried *NA* and *TP5* (group 9). F_2_ females that carried a maternally inherited telomeric element and a paternally inherited *H*(*hsp*/*TP5*)*D* transgene also repressed GD strongly—6.9% GD when the maternally inherited element was *TP5* (group 5) and 13.9% GD when it was *NA* (group 11). However, an *H*(*hsp*/*TP5*)*D* transgene that had been exposed to the presetting effects of either *TP5* (group 6) or *NA* (group 12) did not repress GD at all. Thus, like the diverse non-*TP*s in *M5*; *Birm*, the *TP5* element within the *H*(*hsp*/*TP5*) transgene is not susceptible to presetting by *TP*s even though it can interact genetically with them to enhance cytotype regulation.

We also addressed the reciprocal issue—whether the *H*(*hsp*/*TP5*)*D* transgene could preset a paternally inherited telomeric *TP5* element. In this part of the analysis, *w*; *H*(*hsp*/*TP5*)*D*/+ F_1_ females were crossed to *TP5 w* males to obtain *w/TP5 w*; +/+ females (group 13) and *w/TP5 w*; *H*(*hsp*/*TP5*)*D*/+ females (group 14), which were then test-crossed to Harwich *y w* males. The females of group 13 could reveal if the repression ability of a paternally inherited telomeric *TP5* element is enhanced by a strictly maternal (*i.e.*, presetting) effect of the *H*(*hsp*/*TP5*)*D* transgene, and the females of group 14 could reveal if this ability is enhanced by the combined maternal and zygotic effects of the transgene. Nearly all (>98%) of the daughters from both sets of test crosses were dysgenic. Thus, the repression ability of a paternally inherited telomeric *TP5* element is not enhanced by the maternal or zygotic effects of the *H*(*hsp*/*TP5*)*D* transgene.

## Discussion

*P* elements inserted in the heterochromatic DNA at the XL telomere serve as anchors of cytotype regulation of *P*-element activity in the germ line. The effectiveness of this regulation can be assessed by measuring how well these *TP*s repress hybrid dysgenesis. Genetic analysis using the frequency of GD as the experimental end-point has shown that two *TP*s—either structurally the same or different—establish very strong cytotype regulation in females, whereas a single maternally inherited *TP* represses GD modestly and a single paternally inherited *TP* does not repress GD at all. Cytotype regulation by two *TP*s is therefore interactive rather than additive—that is, the regulatory effect of the two *TP*s is much greater than the sum of their separate effects.

One event contributing to very strong cytotype regulation in females with two *TP*s is the activation of the paternally inherited *TP*. Functionally active and inactive piRNA loci appear to produce the same steady-state levels of sense and antisense transcripts (De Vanssay *et al.* 2012). The activation of a paternally inherited *TP* therefore likely involves a posttranscriptional event that allows its transcripts—or transcripts that contain its sequence—to be processed into *P*-element piRNAs. *P*-element piRNAs synthesized in the mother’s germ line and transmitted through the egg cytoplasm may play a key role in this event, possibly by engaging with the transcripts of the *TP* to generate primary *P*-element piRNAs, or to initiate ping-pong cycling to generate secondary *P*-element piRNAs. In effect, the maternally transmitted *P*-element piRNAs preset the zygote to produce piRNAs from the paternally inherited transcripts of the *TP*. When a maternally transmitted *TP* is also present in the zygote, piRNA synthesis can be augmented by processing transcripts from this element as well, leading to enough *P*-element piRNAs to provide a strong defense against dysgenesis in future offspring. De Vanssay *et al.* (2012) have shown that two *TP*s generate approximately twice as many piRNAs as one maternally inherited *TP*. However, the regulatory effect of two *TP*s is much greater than twice the regulatory effect of a single maternally inherited *TP*. Thus, the strength of cytotype regulation is not simply proportional to piRNA abundance.

Presetting by maternally transmitted *P*-element piRNAs would be expected to play an important role in maintaining cytotype regulation in homozygous *TP* stocks. In each generation, these piRNAs would be needed to jumpstart the production of *P*-element piRNAs from the *TP*s in the genotype. Without a presetting effect, piRNA production would be sluggish and cytotype regulation would be impaired. Presetting also appears to influence the behavior of other piRNA loci. De Vanssay *et al.* (2012) found that an inactive piRNA locus in the middle of chromosome *2*R could be activated by the presetting effect of an active “allele” of this locus, and that the activated locus remained active for many generations. However, the persistence of the active state may have depended on maternal transmission of the locus—and the piRNAs that it produced—over the course of the experiment; that is, the stable expression of piRNAs from this locus may have required the input of maternally transmitted piRNAs each generation.

The abundance and sequence complexity of maternally transmitted piRNAs are likely to influence the effectiveness of presetting. We found that the telomeric elements *TP5*, *TP6*, and *NA* could preset the activation of a paternally inherited *NA* element. Among these three presetting elements, *NA* had the weakest effect, possibly because it came from a heterozygous stock with a diminished ability to generate *P*-specific piRNAs. *TP6* also had a weak presetting effect, but *TP5* had a strong effect. Because *TP5* shares more sequences with *NA* (1384 nucleotides) than *TP6* does (1091 nucleotides), it would be expected to target a more complex array of piRNAs to the transcripts of *NA* and thereby enhance the prospects for these transcripts to be processed into piRNAs. Thus, the greater similarity between *TP5* and *NA* may explain why *TP5* is better able to preset the activation of *NA*.

The strong cytotype regulation that develops in females that carry two *TP*s was impaired by heterozygous mutations in the *aub*, *piwi*, and *Su*(*var*)*205* genes. The *aub* mutations acted in the mothers of these females. Aub protein is located in the nuage, an indistinct region on the cytoplasmic side of the nuclear membrane where ping-pong cycling is thought to take place (Lim and Kai 2007; Kibanov *et al.* 2011; Nagao *et al.* 2011; Zhang *et al.* 2011; Anand and Kai 2012). In *aub^+^/aub*^−^ females, the Aub protein may be depleted to such an extent that ping-pong cycling is impaired, leading to a smaller pool of piRNAs in the eggs of these females—too small, perhaps, to stimulate the production of *P*-element piRNAs from the *TP*s in their daughters. Another possibility is that Aub is involved in the transport of maternal piRNAs. Depletion of Aub may therefore curtail the delivery of piRNAs to the zygote.

The *piwi* mutations acted zygotically to impair cytotype regulation in females with two *TP*s. However, this effect was seen only when one of the *TP*s came from heterozygous *TP*/+ mothers, a condition that would be expected to diminish the abundance of maternally transmitted piRNAs. Piwi is a nuclear protein that may influence chromatin organization, possibly in partnership with HP1, the protein encoded by the *Su*(*var*)*205* gene (Yin and Lin 2007; Wang and Elgin 2011). The limited effect of the *piwi* mutations suggests that the Piwi protein is involved in the activation of *TP*s, perhaps by mediating associations between the presetting piRNAs and the *TP*s (or their transcripts). Genomic analyses have indicated that Piwi has multiple roles in transposon regulation (Le Thomas *et al.* 2013; Rozhkov *et al.* 2013).

The synergism between two *TP*s was profoundly impaired by a heterozygous *Su*(*var*)*205* mutation, no matter whether the mutation was inherited along with the maternal *TP* or with the paternal *TP*. HP1 is found at many chromosomal locations, but mainly in the pericentric and telomeric heterochromatin (James *et al.* 1989). Mutational depletion of this protein might therefore disrupt the organization of heterochromatin. In addition, stocks that are heterozygous for a *Su*(*var*)*205* mutation develop elongated telomeres (Savitsky *et al.* 2002). Together, these epigenetic and genetic changes could impair the production of piRNAs from the *TP*s by disrupting the transcription of the locus in which these elements are inserted, or by preventing maternally transmitted piRNAs from jumpstarting primary piRNA synthesis. Another possibility is that mutational depletion of HP1 interferes with the repressive modification of chromatin in and around *P* elements in the genomes of test-cross offspring, with the result that these elements are mobilized by the P transposase, ultimately causing dysgenesis.

Non-*TP*s interact synergistically with *TP*s to enhance cytotype regulation. The enhanced regulation is as strong as that created by synergism between two *TP*s and is transmitted to offspring independently of either the *TP* or the non-*TP*—that is, as a strictly maternal effect. The synergism between *TP*s and non-*TP*s is impaired by mutational depletion of HP1, Piwi, or Aub (Belinco *et al.* 2009) and is thought to reflect ping-pong amplification of *P*-element piRNAs (Simmons *et al.* 2012). It is much stronger when the *TP* is maternally inherited, presumably because the *TP* comes along with *P*-element piRNAs that jumpstart ping-pong cycling after fertilization. As examples, we found that three different *TP*s interacted strongly with non-*TP*s from the strain Muller-5 Birmingham, and that the two *TP*s tested (*TP5* and *NA*) interacted strongly with the nontelomeric *H*(*hsp*/*TP5*)*D* transgene. Females carrying combinations of these maternally inherited *TP*s and paternally inherited non-*TP*s developed a strong ability to repress hybrid dysgenesis in their progeny. However, their sisters, which carried paternally derived non-*TP*s but did not carry a maternally derived *TP*, failed to develop this ability. This failure indicates that a zygotic effect of the *TP* is needed for the enhancement of cytotype regulation. The strictly maternal effect of the *TP* cannot elicit any regulatory ability from the Birmingham *P* elements or the *H*(*hsp*/*TP5*)*D* transgene—that is, these non-*TP*s are not affected by presetting. After fertilization, the *P*-element piRNAs associated with this maternal effect would be expected to initiate a ping-pong cycle fed by mRNAs transcribed from the paternally inherited non-*TP*s; however, without a *TP* to continue supplying primary piRNAs, this cycle is stymied.

We also found that a paternally inherited *TP* could not be preset by the strictly maternal effect of a non-*TP*. Thus, if RNAs from the non-*TP* are maternally transmitted, then they cannot elicit regulation from a paternally inherited *TP*. However, maternally inherited non-*TP*s can interact with a paternally inherited *TP* to bring about moderate to strong cytotype regulation (Table 4) (Simmons *et al.* 2007a, 2012). A plausible explanation is that as the paternally inherited *TP* begins to generate piRNAs—that is, as it is reactivated—these RNAs drive a ping-pong cycle fed by mRNAs from zygotic expression of the non-*TP*s. A population of secondary piRNAs then develops to regulate *P*-element activity.

Maternally transmitted *P*-element piRNAs play an important role in cytotype regulation. Without them, flies do not develop their full potential to repress hybrid dysgenesis. This arrested development implies that the small RNAs generated from the repetitive DNA of the TAS of *X*L—originally called repeat-associated small interfering (rasi) RNAs—are not so effective in triggering the production of piRNAs from paternally inherited *TP*s. However, these repeat-associated RNAs may be needed to maintain the heterochromatic state of the *X*L telomere. This state may minimize the chance for pairing between the repeated DNA within this telomere and similar DNA sequences at other telomeres. Such pairing could lead to chromosome nondisjunction during meiosis, or to inappropriate recombination. Thus, the repeat associated RNAs may primarily be involved in preventing chromosome entanglements that could lead to aneuploid gametes. However, loci that generate these RNAs clearly have acquired a secondary function: to regulate transposable elements. A transposon inserted into one of these loci is assimilated into a system that generates small RNAs with specificity to the transposon. As our genetic analysis of *P* elements in the TAS of *X*L shows, the entire transposon family then becomes regulated by the system for producing small RNAs. The prior existence of this and other epigenetic systems to maintain chromosomal integrity may be the reason that transposons are tolerated—and abundant—in eukaryotic genomes (Federoff 2012).
